# A technique for parallel query optimization using MapReduce framework and a semantic-based clustering method

**DOI:** 10.7717/peerj-cs.580

**Published:** 2021-06-01

**Authors:** Elham Azhir, Nima Jafari Navimipour, Mehdi Hosseinzadeh, Arash Sharifi, Aso Darwesh

**Affiliations:** 1 Department of Computer Engineering, Science and Research Branch, Islamic Azad University, Tehran, Iran; 2 Future Technology Research Center, National Yunlin University of Science and Technology, Douliou, Yunlin, Taiwan, R.O.C.; 3 Pattern Recognition and Machine Learning Lab, Gachon University, 1342 Seongnamdaero, Sujeonggu, Seongnam, Republic of Korea; 4 Department of Information Technology, University of Human Development, Sulaymaniyah, Iraq

**Keywords:** Query optimization, Access plan recommendation, Cluster computing, Parallel Processing, MapReduce, DBSCAN Algorithm

## Abstract

Query optimization is the process of identifying the best Query Execution Plan (QEP). The query optimizer produces a close to optimal QEP for the given queries based on the minimum resource usage. The problem is that for a given query, there are plenty of different equivalent execution plans, each with a corresponding execution cost. To produce an effective query plan thus requires examining a large number of alternative plans. Access plan recommendation is an alternative technique to database query optimization, which reuses the previously-generated QEPs to execute new queries. In this technique, the query optimizer uses clustering methods to identify groups of similar queries. However, clustering such large datasets is challenging for traditional clustering algorithms due to huge processing time. Numerous cloud-based platforms have been introduced that offer low-cost solutions for the processing of distributed queries such as Hadoop, Hive, Pig, etc. This paper has applied and tested a model for clustering variant sizes of large query datasets parallelly using MapReduce. The results demonstrate the effectiveness of the parallel implementation of query workloads clustering to achieve good scalability.

## Introduction

Today, IT and distributed environments have facilitated whatever you can imagine: communication, managing, and businesses. The critical goal of distributed environments is to deliver remote services residing at disparate sites to users. As a novel form of distributed environment, the goal of cloud technology is to provide remote services with high dependability, dynamicity, and scalability using virtualization technology (Buyya, Broberg & Goscinski, 2010; Ebadi & Jafari Navimipour, 2019; Cheng, 2021; Angela Jennifa Sujana, Revathi & Joshua Rajanayagam, 2020). Cloud technology provides numerous kinds of virtualized services such as storage services, healthcare services, operating systems, and networks; it draws upon distributed computing concepts to offer consumers on-demand services (Vivekrabinson & Muneeswaran, 2021; Sharma & Kalra, 2019).

In large-scale distributed databases such as distributed cloud databases, the query optimization issue can not be solved efficiently (Singh, 2016; Han, Youn & Lee, 2017; Panahi & Navimipour, 2019). The cost model of a distributed query involves local computing costs and node-to-node communication ones. Therefore, the space of alternative execution plans can become large, depending on the query’s complexity and the opportunity to execute subqueries at any node. There are many execution plans, each with a corresponding cost for a particular query. Therefore, the query optimizer needs to find an optimal plan with minimum cost. However, the optimizer cannot search the space of all possible execution plans efficiently. The access plan recommendation approach has been presented to reduce the query optimization costs by reusing execution plans produced for previous queries (Ghosh et al., 2002; Zahir & El Qadi, 2016; Zahir, El Qadi & Mouline, 2014). The query optimizer uses the pre-executed query plans to execute new incoming queries that are similar to previous queries in the proposed approach.

The primary goal of the access plan recommendation approach is to reuse the previous query plans based on the likeness of query statements. However, clustering such large datasets is a challenge for traditional clustering algorithms due to its huge processing times. This can be addressed by using MapReduce, a scalable and distributed processing technique (Shabestari et al., 2019). It is a programming paradigm used for parallel distributed processing of large datasets (Dean & Ghemawat, 2008). Query clustering involves large datasets for which MapReduce is an attractive means to reach a solution with high-quality results within an acceptable amount of time. This paper presents a scalable model for the access plan recommendation approach using MapReduce. The Term Frequency (TF) method (Makiyama, Raddick & Santos, 2015) and cosine measure are applied with a feature representation of Structured Query Language (SQL) to detect the similarity of the queries. The article also employs the popular density-based clustering technique Density-Based Spatial Clustering of Applications with Noise (DBSCAN) (Ester et al., 1996). Finally, the MapReduce technique is used to parallelize the query preprocessing and clustering operations. The specific contributions of this paper are:

(i) Implementing an efficient access plan recommendation method for query plan recommendation using a MapReduce workflow.
(ii) Evaluating the proposed approach on multiple-query datasets based on different structures.

The following classification will be discussed in the rest of the paper. In “Overview and background”, the DBSCAN clustering algorithm and the MapReduce model are introduced. Previous studies are reviewed in “Related work”. The next section presents the introduced method for query plan recommendation in the distributed systems. The section related to the “Results” illustrates the simulation outcomes. In “Discussion”, the findings of this study are discussed. Finally, the last section summarizes the paper and offers some indications for the upcoming studies.

## Overview and background

Clustering is a technique to group some related objects using similarity or location information (Sadrishojaei et al., 2021). Partitioning, hierarchical (Solihah, Azhari & Musdholifah, 2020), grid, density (Mehdi Cherrat, Alaoui & Bouzahir, 2020), graph, and model-based algorithms are different clustering methods. The performance of these methods is assessed by several factors such as the number of input parameters, data size, cluster shapes, and noise. Moreover, considering the fast growth of IT and the huge data generated daily by millions of people (Rahmani et al., 2021), there is no doubt that a single device may not be able to handle such quantities of data. So, novel technologies are needed to store and extract information.

First, the DBSCAN clustering method is proposed in the “Density-based Clustering Algorithm”. Then, the MapReduce workflow is briefly introduced to process large datasets with parallel distributed algorithms in “MapReduce Overview”.

### Density-based clustering algorithm

DBSCAN is a key method for clustering unlabeled datasets. It is a density-based clustering method that can generate random shape clusters. The main idea is to generate a cluster from any point with a minimum number of points inside an assumed radius. A few crucial descriptions of DBSCAN are Ester et al. (1996):

• **Eps neighborhood:** The *Eps* neighborhood of a point *p*, expressed as *N*_*Eps*_(*p*) = {*q* ∈ *D*|*dis*(*p*, *q*) ≪ *Eps*}, where *D* contains points within distance *Eps* from point *p*.
• **Directly density-reachable:** *p* is directly density-reachable from *q* if *p* is in the *Eps* neighborhood of *q* (i.e.,  *p* ∈ *N*_*Eps*_(*q*)) and *q* is a core point (i.e.,  |*N*_*Eps*_(*q*)| ≫ *Minpts*).
• **Density-reachable:** If a set of points exists $ \left\{ {r}_{i}{|}i=0,\ldots ,n \right\} $ where each *r*_*i*_ is directly density-reachable from *r*_*i*+1_, then *r*_*i*_ is density-reachable from a point *o* where *o* ∈ {*p*_*j*_|*j* = *i* + 1, …, *n*}.

The algorithm begins by picking a random point *p*, then retrieving all density-reachable points from *p*. If *p* is a core point, the algorithm creates a cluster. If *p* is a border point and none of the points is density-reachable from *p*, then the algorithm picks the next point of the dataset. This procedure is repeated for all points.

### MapReduce overview

**Figure 1:**
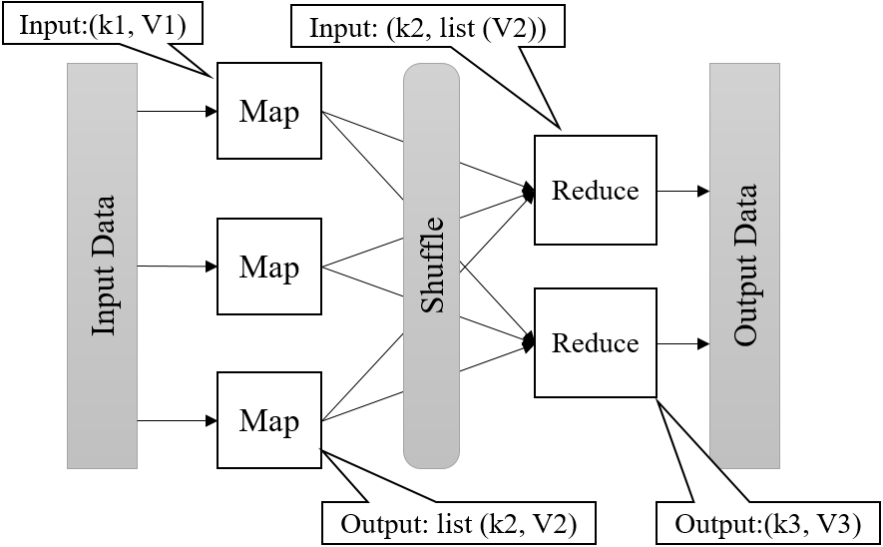
Data flow in MapReduce.

MapReduce (Dean & Ghemawat, 2008) is a model for large-scale and distributed data processing. MapReduce can be scaled to thousands of nodes. The input data is divided into smaller chunks and kept on a distributed file system. In MapReduce, data should be indicated as (key, value) pairs. Figure 1 shows the MapReduce data flow. Map, Shuffle, and Reduce are three main phases of MapReduce (White, 2012). The map function takes the input pair (k1,v1) and produces one or more intermediate key/value pairs (k2, v2). The shuffle stage partitions the intermediate pairs and sends them to reduce functions. The “reduce function” groups pair values with an identical key (k2, list(v2)) and generate the ultimate output pair list (k3, v3) for all of them (Khezr & Navimipour, 2015).

### Related work

Most studies have focused on query optimization methods for various systems due to the significance of the query optimization problems. In this section, access plan recommendation mechanisms are studied to optimize the query.

Query optimization is a method to provide low-cost answers to queries. The purpose of a query optimizer is to evaluate alternative query plans to determine the best query plan. The major query optimization techniques can be divided into seven main classes in the cloud: cost-based query plan enumeration, agent-based, schema-based, security-based, Multiple Query Optimization (MQO), adaptive query optimization, and access plan recommendation (Azhir et al., 2019b; Azhir et al., 2019a). The access plan recommendation mechanisms (Ghosh et al., 2002; Zahir & El Qadi, 2016; Zahir, El Qadi & Mouline, 2014) use similarity-based machine learning methods to recommend an old query execution plan to the optimizer. Here we describe work related to query optimization using access plan recommendation mechanisms.

Azhir et al. (2021) proposed an automatic hybrid query plan recommendation method based on incremental DBSCAN and NSGA-II. Dunn and Davies–Bouldin indices were used to evaluate the goodness of clusters. The results of the proposed algorithm were compared to incremental DBSCAN and K-means. According to the experimental results, the introduced algorithm outperforms the other well-known approaches in terms of accuracy.

Ghosh et al. (2002) developed a plan recycling tool, named Plan Selection Through Incremental Clustering (PLASTIC), to cluster queries using their structures and statistics. The PLASTIC was developed based on the Leader clustering algorithm presented by Hartigan (1975). Based on the trial outcomes, the proposed tool can predict the right query plan in the majority of cases. Furthermore, according to the experimental results, high precision, short time, and low space overhead are the advantages of PLASTIC. To enhance the usability of PLASTIC, Sarda & Haritsa (2004) improved the queries’ feature vector. Also, a decision-tree classifier is included in PLASTIC for effective cluster assignments.

An efficient query plan recommendation method was introduced by Zahir, El Qadi & Mouline (2014). The main aim of this approach is to reuse earlier query plans in executing future queries. The method identifies the likeness between the queries (Makiyama, Raddick & Santos, 2015; Aligon et al., 2014; Aouiche, Jouve & Darmont, 2006; Kul et al., 2018). The clustering technique is grounded on K-Means and Expectation-Maximization (EM) methods. The similarity of the queries was identified using the SQL queries semantics. The method can decrease the query optimization cost.

Finally, Zahir & El Qadi (2016) introduced a query plan prediction technique by identifying the similarity among the statements of the queries. Some primary classification techniques like Support Vector Machine (SVM), Association Rule (AR), and Naive Bayes (NB) were applied in this research. The outcomes revealed that the AR can offer more precise forecasts than SVM and NB techniques.

## Methods

We have demonstrated the recommendation process on a simple random selection query *Q*_*n*_.*Q*_*n*_ is defined as:

***SELECT*** *course_id, title*

***FROM*** *course*

***WHERE*** *course.dept_name* =* ’comp. sci.’;*

Results of similarity calculation have shown that the highest similarity has been detected with a certain query *Q*_3_ defined as:

***SELECT*** *course. course_id, course. title*

***FROM*** *course*

***WHERE*** *dept_name* =* ’?’;*

Hence, we have recommended the optimizer use the access plan of *Q*_3_ to execute query *Q*_*n*_.

In “Access Plan Recommendation”, we present the access plan recommendation method for query optimization in detail. “Parallel Access Plan Recommendation” develops a parallel similarity-based query optimization approach to decrease the query optimization cost, using the MapReduce programming model.

### Access plan recommendation

The main purpose of the access plan recommendation approach is to reuse previously-used execution plans for new queries. It examines the textual similarity between the new incoming query and the earlier queries to use previously executed query plans for new queries. This method represents query statements as feature vectors and then compares them to compute the similarity between them. The query optimizer uses an old query plan for a new query if a similarity exists between them. Therefore, the proposed approach reduces the required cost of producing a new access plan for new incoming queries.

**Figure 2:**
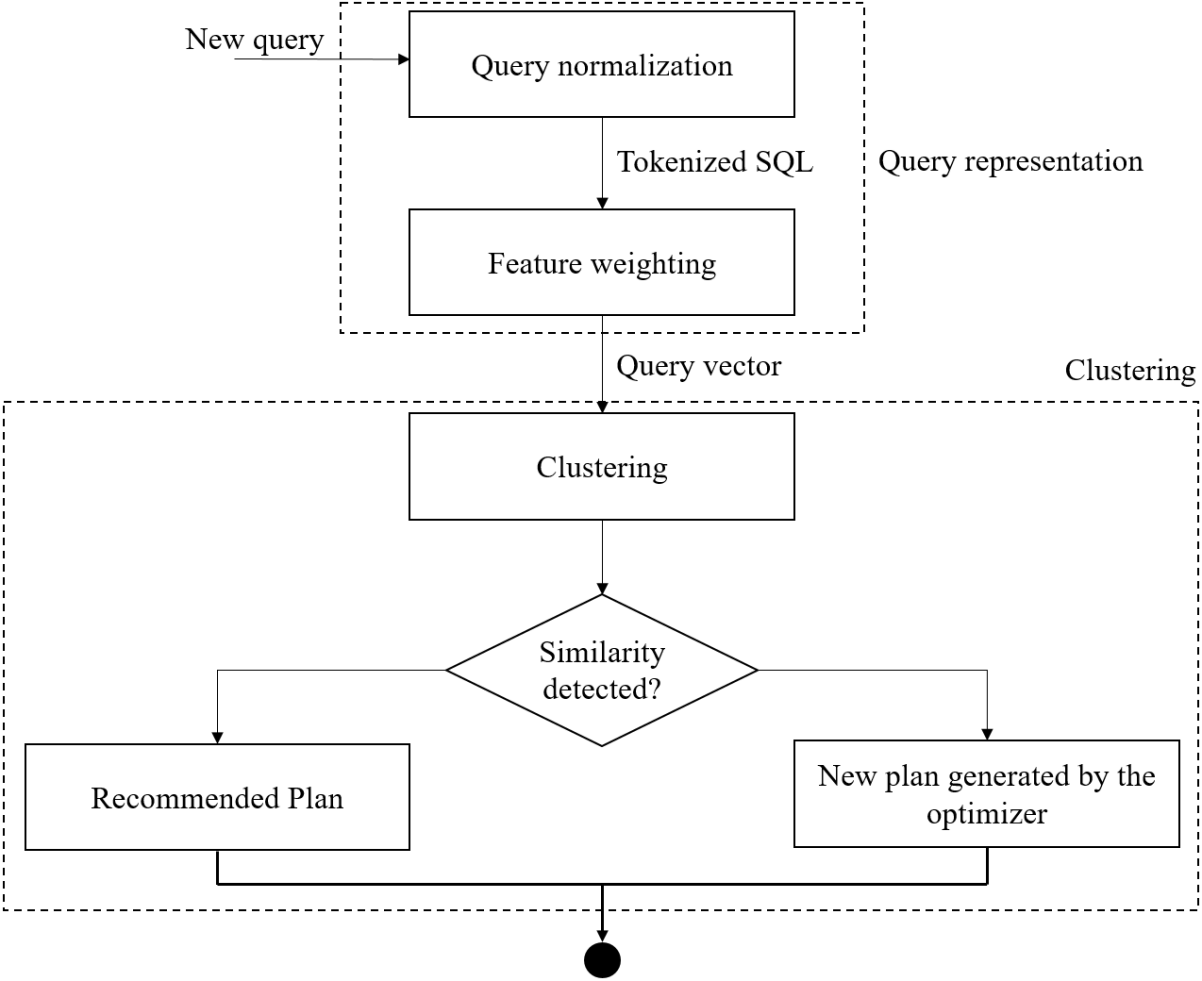
The access plan recommendation approach.

Figure 2 shows the access plan recommendation approach to optimize the query. The R DBSCAN package (Hahsler, Piekenbrock & Doran, 2019) is used to group the queries in different clusters. As shown in Fig. 2, query representation and clustering are the two main steps of the presented approach. In the query representation step, a tokenizer breaks the query text into tokens. Then, a weight is assigned to each token. Query normalization and feature weighting are performed in this phase. The assignment of queries to clusters and the recommendation of the access plans are done in the clustering phase.

Cosine similarity (Nguyen & Amer, 2019) and Jaccard coefficient (Jaccard, 1912) are the two most popular similarity measures used for text documents (Huang, 2008). The performance of these measures has been assessed through empirical experiments. As a result, the Cosine similarity is used to identify similar queries.

The cosine measure calculates the similarity between two vectors by calculating the cosine of the angle created by two vectors. The Cosine similarity is calculated by Eq. (1) (Nguyen & Amer, 2019).

(1) \begin{eqnarray*}\cos \nolimits \left( \Theta \right) = \frac{\sum _{k=1}^{n}{x}_{1k}{x}_{2k}}{\sqrt{\sum _{k=1}^{n}{x}_{1k}^{2}}\sqrt{\sum _{k=1}^{n}{x}_{2k}^{2}}} \end{eqnarray*}

Furthermore, the Jaccard coefficient is applied to calculate the similarity of two queries based on the presence or absence of features. It is computed by dividing the total number of mutual features between two queries by the total number of features in at least one of the two queries. It is denoted as Jaccard (1912):

(2) \begin{eqnarray*}J= \frac{{|}A\bigcap B{|}}{{|}\mathrm{A}\bigcup B{|}} \end{eqnarray*}

The similarity value is between 0 and 1. If the value is 1, the two queries are identical.

### Parallel access plan recommendation

In this section, the primary scheme for parallel access plan recommendation is presented. In this regard, the parallel parts are analyzed, and how the needed processes can be formalized as map/reduce procedures is fully described.

**Figure 3:**
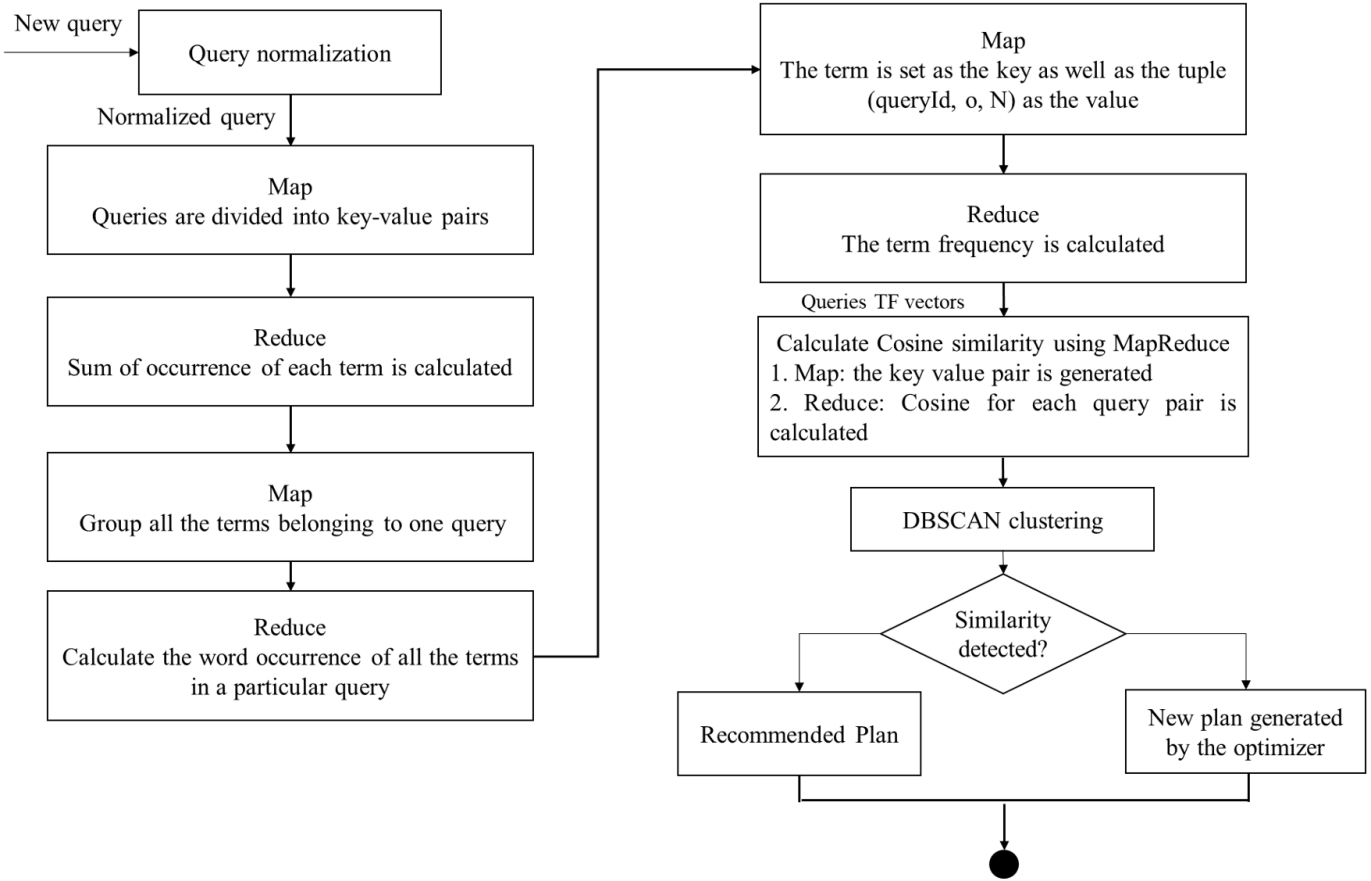
The parallel access plan recommendation using MapReduce flow.

As shown in Fig. 3, the input of the clustering method is the weight matrix of the queries to access plan recommendation. Each row indicates a query vector, and each value in the row specifies the weight of a feature. The main stage of the clustering procedure is the calculation of the distance of query vectors. Therefore, each query is calculated for similarity so that clustering can be easy. In this paper, the queries have been clustered using TF values and distance measured using Cosine distance.

As mentioned, the preparation of query vectors and the computation of Cosine similarity between the generated vectors can be performed in parallel with Map functions and Reduce functions in 4 stages to perform the clustering. The details of Map functions and Reduce ones are as follows (Fig. 3):

The JSQL parser (http://jsqlparser.sourceforge.net/) parses the SQL statements and provides the ability to manipulate them. First, a custom JSQL parser library and various standardization rules are employed to improve clustering quality (Azhir et al., 2021). The rewriting module parses the queries’ text to eliminate string literals, constants, temporary names of tables and columns, syntactic sugar, and database namespaces (Kul et al., 2018). In addition, the parser attempts to tokenize the query and qualify each token with the select, from, group-by, and order-by statements.

1. In the first step, the occurrences of each token in every query are calculated:
  • Map: in the Map phase, each query is converted into a key-value form. In this phase, the term and queryID are chosen as the key. Also, the number 1 is assigned to each term as the value.
  • Reduce: here, the sum of occurrences of each term is computed. The key is set as the term and queryID and the value is set as the number of occurrences.
2. In the second stage, the produced features are weighted according to their frequency. Therefore, the total number of terms of each query can be calculated by:
  • Map: all the terms of each query are grouped in this step. The queryID is assigned as the key and the term. The occurrence of each term in every query is assigned as the value.
  • Reduce: here, the sum of terms in each query is counted. The key-value sets are returned with the tuples (queryID, N) as the key, and the tuples (term, o) are returned as the value, where o is the total occurrence of each term in every query, and N is the sum of terms in the query.
3. In the third step, the frequency of each term in a query is computed. The TF method evaluates the term’s or phrase’s importance in an assumed query. This measure can be computed by Eq. (3) (Makiyama, Raddick & Santos, 2015).
  (3) \begin{eqnarray*}tf(q,f)\text{: Frequency of feature}f\text{in query}q;\end{eqnarray*}
  • Map: here, the term is set as the key and the tuple (queryID, o, N) as the value.
  • Reduce: the term frequency is calculated.
4. In the final stage, the Cosine similarity of query vectors is computed (Victor, Antonia & Spyros, 2014).
  • Map: in the Map phase, the key/value pair is produced. The query IDs are set for the key, and the term frequency vectors are set for the value.
  • Reduce: here, the Cosine similarity for each query pair is computed.

At last, the clustering segmentation of DBSCAN is performed based on the Cosine similarity results.

## Results

Here, the effectiveness of the introduced method is assessed to solve the query access plan problem. Also, several experiments are performed to assess the proposed parallel approach. The performance metrics and experiments are described in this section.

The experiments in this study are performed on a Hadoop cluster with various numbers of virtual machines (nodes). The environment was configured on a physical machine with a 2.8 GHz Intel Core i7 processor with 16 GB of memory. Table 1 shows a summary of the arrangement of the Hadoop clusters. Hadoop 2.8.0 was installed, and necessary adjustments were made on each node. One of the nodes was selected as master, and the other nodes were selected as workers.

**Table 1:** The Hadoop cluster setup.

Nodes	OS	Configurations	No. of cores	Processor base frequency	Configuration
Master machine (Name node)	Red Hat (64bit)-Linux	8 GB RAM	4	2.53 GHZ	Intel Core i7
Slave machines (Data nodes)	Red Hat (64bit)-Linux	3 GB RAM	2	2.53 GHZ	Intel Core i7
Hadoop version		Hadoop-2.8.0	–		
Virtual machine management		Virtualbox 6.1.16			

A query template shows a query where the bind variables have substituted several constants (or all of them). The IIT Bombay dataset (Chandra et al., 2015) is used to generate 5121 queries in fifteen query sets with diverse structures like simple selection query logs, selection/ join query logs, and query logs consisting of selection/join and nested sub-queries. In addition, various values are allocated to the bind variables. Table 2 shows the nature of the generated SQL datasets.

**Table 2:** The description of datasets.

Features	Dataset name	No. of classes	No. of individuals
Selection	S1	3	95
	S2	7	235
	S3	10	389
	S4	12	481
	S5	14	593
Selection/join, from, group-by and order-by	SJ1	4	60
	SJ2	7	199
	SJ3	10	389
	SJ4	12	481
	SJ5	14	593
Selection/join, from, group-by and order-by alongsidenested sub-queries	SJN1	4	108
	SJN2	6	140
	SJN3	9	325
	SJN4	11	440
	SJN5	14	593

When a query is performed in the Oracle database, a unique value is assigned to its query plan. To determine the real clusters, each set of queries is categorized manually using the unique value of their execution plans. As shown in Table 2, SJN5 contains 593 queries, including subqueries with diverse execution plans in 14 classes.

### Performance metrics

It is problematic to state when a clustering result can be suitable. So, many clustering validation methods have been presented. The introduced technique’s results are confirmed using Dunn Index (DNI), Silhouette Coefficient, and the Adjusted Rand Index (ARI).

The Dunn Index calculates the smallest distance among clusters and the largest interval between data objects from a similar cluster. It recognizes compressed and distinct clusters. The Dunn index is defined by Eq. (4) (Zaki, Meira Jr & Meira, 2014):

(4) \begin{eqnarray*}{D}_{k}=mi{n}_{i=1,\ldots ,k} \left\{ mi{n}_{j=i+1,\ldots ,k}( \frac{d({c}_{i},{c}_{j})}{ma{x}_{r=1,\ldots ,k}diam({c}_{r})} ) \right\} \end{eqnarray*}

where *d*(*c*_*i*_, *c*_*j*_) is the dissimilarity between clusters. *c*_*i*_ and *c*_*j*_ are defined as $d \left( {c}_{i},{c}_{j} \right) =mi{n}_{x\in {c}_{i},y\in {c}_{j}}(d \left( x,y \right) ).diam(c)$ is the diameter of the cluster. It is defined as $diam(c)=ma{x}_{x,y\in c}(d \left( x,y \right) )$.

The Silhouette Coefficient (Rousseeuw, 1987) is another approach to assess the clustering. This index considers cohesion and separation in measuring the quality of clustering. The Silhouette Coefficient is defined as:

(5) \begin{eqnarray*}s \left( i \right) = \frac{b \left( i \right) -a(i)}{\max \nolimits \{a \left( i \right) ,b \left( i \right) \}} \end{eqnarray*}

where for each point *i*, $a \left( i \right) $ indicates the average distance between the point and other points within a similar cluster, and *b*(*i*) is the minimum value concerning all other clusters.

Rand Index (RI) calculates the similarity of two solutions. This index has been designed to exploit the similarity of the partitioning with their original class labels. The RI is between 0 and 1. When two partitions are consistent, the *RI* reaches 1. This index can be calculated by Yeung & Ruzzo (2001):

(6) \begin{eqnarray*}\mathrm{RI}= \frac{\mathrm{a}+\mathrm{d}}{\mathrm{a}+\mathrm{b}+\mathrm{c}+\mathrm{d}} \end{eqnarray*}

where,

• a: two data objects in both partitions are allocated to a similar cluster,
• b: two dissimilar data objects are allocated to the similar cluster,
• c: two similar data objects are allocated to different clusters,
• d: two different data objects are allocated to different clusters.

Hubert and Arabie Zaki, Meira Jr & Meira (2014) presented the ARI measure to enhance RI. We suggest ARI for measuring the correspondence between the two partitions. The ARI is calculated using Eq. (7).

(7) \begin{eqnarray*}\mathrm{ARI}= \frac{ \left( {n\atop 2} \right) \left( \mathrm{a}+\mathrm{d} \right) - \left[ \left( a+b \right) \left( a+c \right) +(c+d)(b+d) \right] }{{ \left( {n\atop 2} \right) }^{2}- \left[ \left( a+b \right) \left( a+c \right) +(c+d)(b+d) \right] } \end{eqnarray*}

## Experiments

In the first step, diverse methods are investigated to measure the similarity of queries. The performance of various similarity measurements has been evaluated by comparing the generated outcomes to those of the original partitions. Given a group of queries categorized according to their plan’s hash values, it is important to comprehend how well different measures can group queries (having identical plan’s hash values) and separate those with dissimilar plan’s hash values. The Jaccard and Cosine indexes are measures of similarity often used to measure the similarity among documents by comparing their feature vectors.

The experiments are applied to query clustering on some query input sets with diverse query structures. After standardizing and generating query vectors, the pairwise distance matrix among each query pair has been calculated using Cosine and Jaccard similarity measures. These two measures’ effectiveness has been assessed via a pairwise distance matrix of feature vectors and a query dataset specified with the real plan’s hash values. Each similarity measure has been evaluated using its consistency with the real cluster labels. The Dunn index and Silhouette coefficient clustering validation methods have been used to recognize the efficiency of a similarity measurement in a different set of queries.

**Figure 4:**
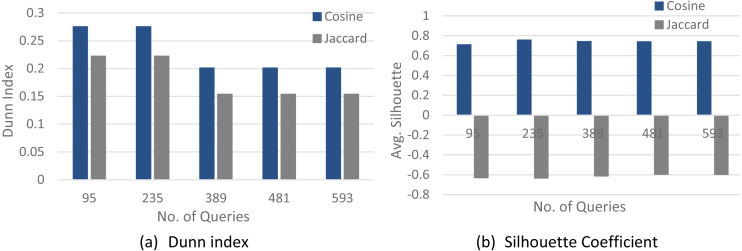
The clustering quality validation for the selection queries datasets (S1–S5). (A) Dunn index; (B) Silhouette coefficient.

**Figure 5:**
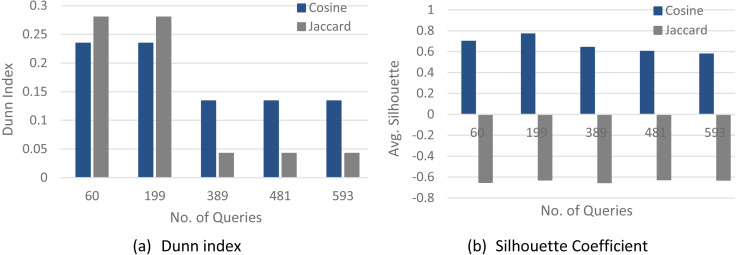
The clustering quality validation for the selection/join queries datasets (SJ1–SJ5). (A) Dunn index; (B) Silhouette coefficient.

**Figure 6:**
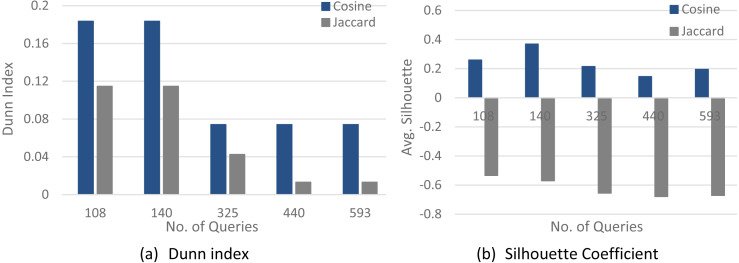
The clustering quality validation factor for the selection/join/nested sub-queries datasets (SJN1–SJN5). (A) Dunn index; (B) Silhouette coefficient.

Figures 4–6 show a comparison between two quality measures for the selection query logs (S1–S5), the selection/join queries (SJ1–SJ5), and the selection/join alongside nested sub-queries (SJN1–SJN5). The low average Silhouette coefficients and Dunn index have been considered incorrect clustering directly affecting the ground-truth quality.

As shown in Figs. 4–6, on most datasets, Jaccard measures have made the Dunn index and average Silhouette coefficient measure worse. However, the Cosine measure seems to work best with the average Silhouette coefficient measure and Dunn index. Therefore, it is concluded that the proposed algorithm will give the same estimated number of clusters with Cosine similarity, which is identical to the real number of clusters.

**Figure 7:**
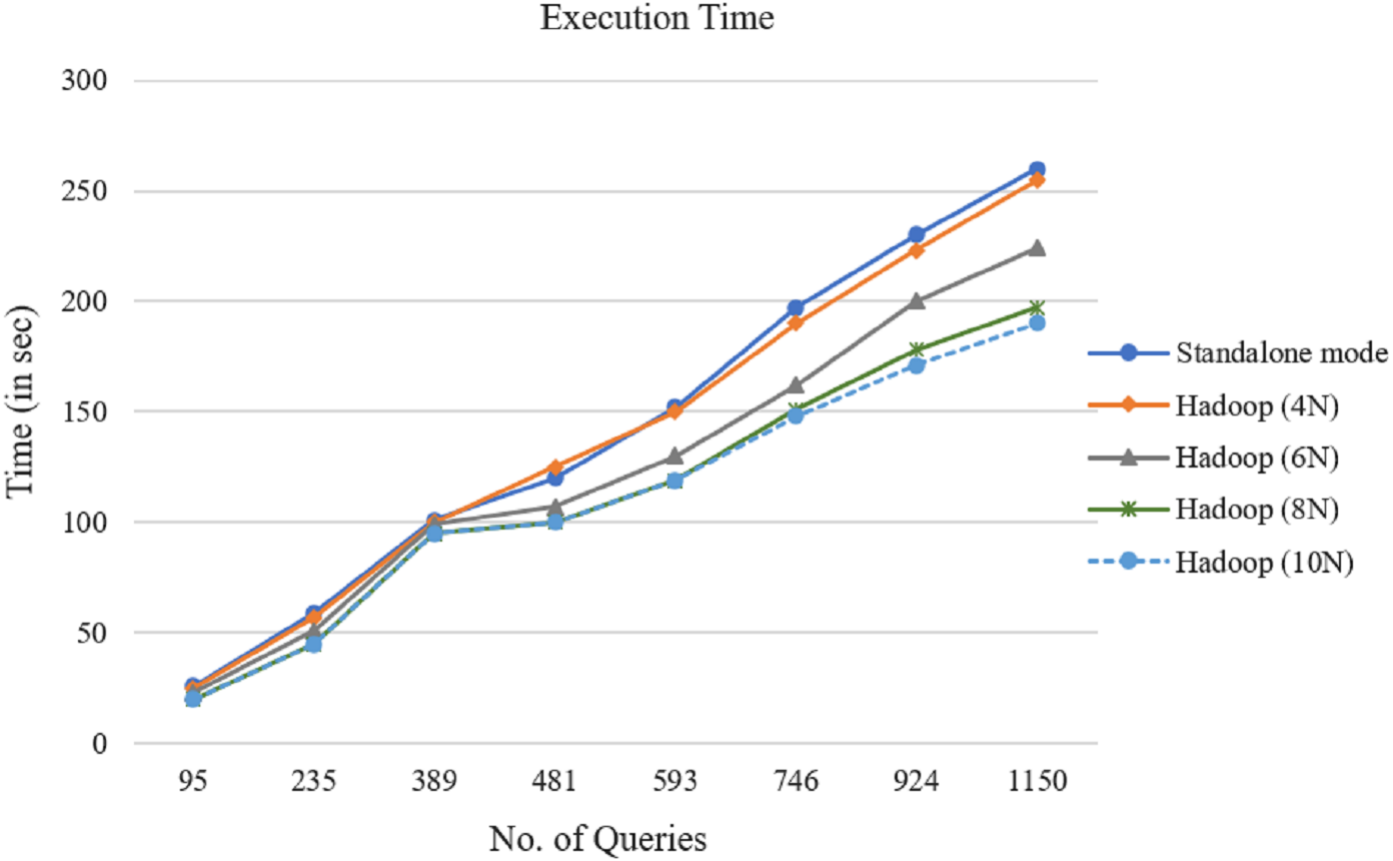
MapReduce processing time variation for the selection queries’ datasets (S1–S5).

In the next step, the proposed parallel algorithm has been applied to the representations of the produced query datasets of Table 2 with the Cosine similarity measure. Figure 7 shows the introduced method’s execution time for different data nodes with varying numbers of queries. Figure 7 shows that when the number of queries increases, the introduced technique’s execution time increases, too. It was found that 4, 6, 8, and 10 Hadoop node always outperform the standalone implementations.

Lastly, the clustering segmentation of DBSCAN was carried out based on the Cosine similarity results. Several experiments have been conducted with variable *Epsilon* (Eps = 0.02, …, 0.18) in steps of 0.02. The *Minpts* is =0, …, 19.

**Figure 8:**
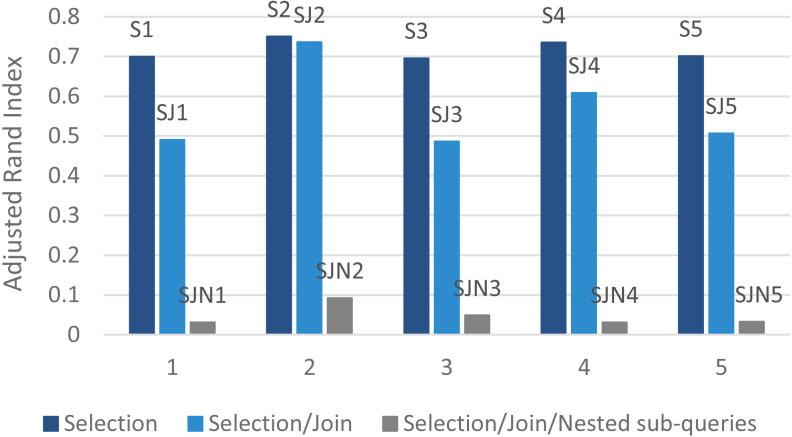
The clustering quality validation using ARI.

Figure 8 and Table 3 present the proposed algorithm’s results using *ARI* (Eq. (7)). The table indicates values for *Eps* and *MinPts* for different datasets to reach the ideal performance. The presented DBSCAN had higher accuracy in the selection queries’ dataset. For example, in the selection queries’ dataset (*k* = 14), *Eps* = 0.12 and *Minpts* = 16 satisfies the ground-truth clustering accuracy. Table 3 indicates that as the complexity and the number of queries increase, clustering efficiency decreases.

**Table 3:** Epsilon and Minpts best values for the proposed algorithm.

Dataset	ARI	No. of Clusters	Minpts	Eps
**Selection queries log**				
S1	0.6997	3	8	0.03
S2	0.7504	7	16	0.1
S3	0.6956	10	16	0.08
S4	0.7348	12	16	0.12
S5	0.7011	14	16	0.12
**Selection/join queries log**				
SJ1	0.4905	1	8	0.06
SJ2	0.7361	5	8	0.06
SJ3	0.4859	12	8	0.09
SJ4	0.6084	12	8	0.06
SJ5	0.5062	14	8	0.09
**Selection/join with nested sub-queries log**				
SJN1	0.0310	2	12	0.06
SJN2	0.0923	2	12	0.06
SJN3	0.0482	3	12	0.02
SJN4	0.0310	5	12	0.06
SJN5	0.0324	7	12	0.06

## Discussion

It is difficult to solve the query optimization problem in large-scale and distributed databases. Unfortunately, the optimizer’s complexity grows with the increase in the number of relations and joins. In this regard, the query optimizer needs to investigate the large space of possible query plans to produce optimal query execution plans. Recommendation-based approaches are developed to help the query optimizer recognize the similarity between old and upcoming ones. This paper presents an efficient MapReduce-based parallel processing method to improve the time-efficiency of the access plan recommendation approach to optimize the query.

In a Hadoop cluster, the MapReduce algorithm was executed with different query datasets. The experiment results demonstrated that the presented parallel access plan recommendation approach could deal with large query logs and improve time efficiency. After comparing the curves in Fig. 7, it is clear that as the number of queries increases, the distance among the curves increases, as well. So the parallel computing efficiency is higher. Particularly with the growing number of queries, this benefit becomes clearer. The results show that the speedup factor increases alongside the number of queries in the MapReduce framework.

On the other hand, as shown in Fig. 8, the proposed algorithm can find the real number of clusters for the selection queries’ dataset. On this basis, it is concluded that the parallel access plan recommendation approach on the Hadoop platform can tackle the large-scale selection queries with higher accuracy and acceptable processing time.

## Conclusions

This paper aimed to improve semantic-based query clustering efficiency in the access plan recommendation regarding the recommendation time. In this paper, we assessed semantic similarities among queries. First, we normalized the query semantics and calculated token occurrences to make the query vectors. Then, we used the access plan recommendation workflow with Cosine measure to produce the queries’ weight matrix. Also, we sped up the clustering process using the MapReduce parallel programming model. The speedup factor increases linearly with the number of Hadoop nodes and dataset size. We obtained the highest speedup of 1. 36 × using 10 Hadoop nodes over the standalone implementation.

It is suggested that the efficiency of other clustering algorithms and query representations methods be examined for plan recommendations in future work. Also, further experiments will be useful to investigate the performance of the proposed technique in various datasets.

##  Supplemental Information

10.7717/peerj-cs.580/supp-1 Supplemental Information 1 Selection queries dataset

10.7717/peerj-cs.580/supp-2 Supplemental Information 2 Codes

10.7717/peerj-cs.580/supp-3 Supplemental Information 3 Selection/join, from, group-by and order-by queries dataset

10.7717/peerj-cs.580/supp-4 Supplemental Information 4 Selection/join, from, group-by and order-by alongside nested sub-queries dataset

## Additional Information and Declarations

### Data Availability

The following information was supplied regarding data availability: We used the Apache Mahout open-source library for creating the TF vectors and calculating pairwise similarities using cosine distance. The associated codes and query datasets are available as Supplemental Files.
